# Supplementary material for: Overcoming laser phase noise for low-cost coherent optical communication
Source: Nat Commun. 2024 Jul 27;15:6339. doi: 10.1038/s41467-024-50439-1 (PMC11283498; doi:10.1038/s41467-024-50439-1)
Supplement: Supplementary file 1 — Supplementary Information [file 41467_2024_50439_MOESM1_ESM.pdf]

Supplementary Materials for
"Overcoming laser phase noise for low-cost coherent optical communication"

Xiansong Fang[1,#], Yixiao Zhu[2,#,†], Xiang Cai[1], Weisheng Hu[3], Zhixue He[3], Shaohua Yu[1,3], and Fan Zhang[1,3,‡]

[1]State Key Laboratory of Advanced Optical Communication Systems and Networks, Frontiers Science Center for Nano-optoelectronics, School of Electronics, Peking University, Beijing 100871, China

[2]State Key Laboratory of Advanced Optical Communication Systems and Networks, Department of Electronic Engineering, Shanghai Jiao Tong University, Shanghai 200240, China

[3]Peng Cheng Laboratory, Shenzhen 518055, China

[#]These authors contributed equally to this work

Corresponding authors: [†]yixiaozhu@sjtu.edu.cn, [‡]fzhang@pku.edu.cn.

(Dated: July 5, 2024)

CONTENTS

Note 1 - Laser Linewidth Measurement Results 2

Note 2 - Single-channel Experimental Setup and DSP Stacks for PS-256-QAM Transmission 3

Note 3 - Detailed Simulation Setup for OSNR Penalty Comparison 5

Note 4 - Detailed Experimental Setup and DSP Stacks for 512-QAM Analog Radio-over-fiber Transmission 6

Note 5 - The Theoretical Dependence of Effective OSNR on the CSPR 8

Note 6 - The Transmission Performance with Different Source Entropy 9

Note 7 - Comparison Between RCM and Time-domain or Frequency-domain Pilot Based Phase Recovery 10

Note 8 - Linear and Nonlinear Equalization Performance in RCM 13

Note 9 - Polarization Distortion and Its Influence on RCM 14

Note 10 - Bias Control Method for Generating the Residual Carrier 16

References 16

NOTE 1 - LASER LINEWIDTH MEASUREMENT RESULTS

We measured the laser linewidth of the 1-MHz and 3-MHz DFB lasers with the delayed self-heterodyne interferometer (DSHI) method [1]. For the DSHI method, we use the Lorentzian curve fitting to get the measured full width at half maximum (FWHM) of the received power spectrum. The desired laser linewidth can be obtained as half of this FWHM value.

As illustrated in Fig. **S1**, for the 3-MHz DFB used in the experiment, the measured precise linewidth is 2.90 MHz (=5.80/2 MHz). Similarly, for the 1-MHz DFB used in the experiment, the measured precise linewidth is 1.325 MHz (=2.65/2 MHz).

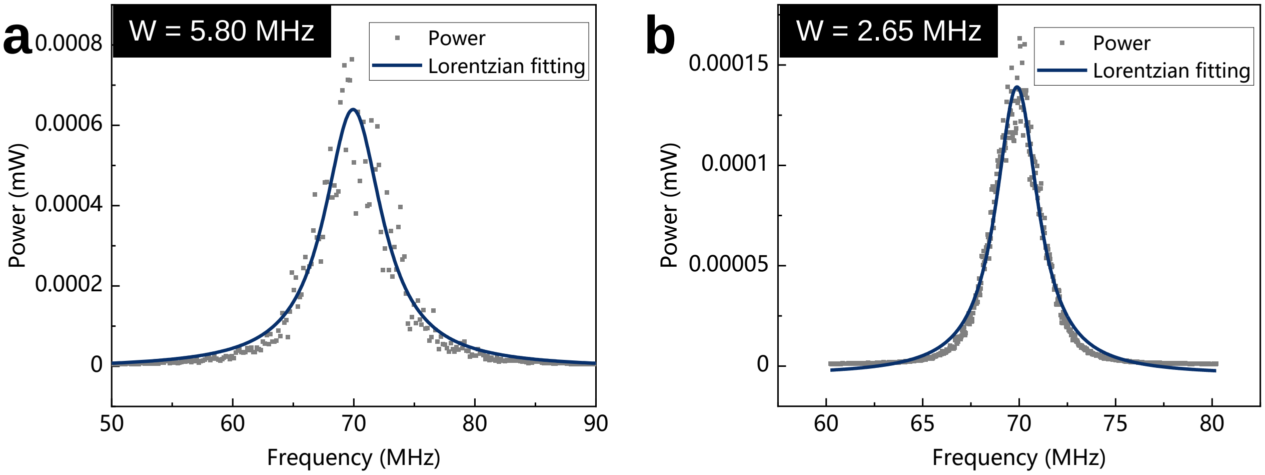

Fig. S1. The measured DSHI power spectra and their Lorentzian curve fitting results of the two DFB lasers. **a** The measured results for the nominal 3-MHz laser. From the Lorentzian curve fitting results, a full width at half maximum (FWHM) of 5.80 MHz is obtained. **b** The meausred results for the nominal 1-MHz laser. From the Lorentzian curve fitting results, a FWHM of 2.65 MHz is obtained.

NOTE 2 - SINGLE-CHANNEL EXPERIMENTAL SETUP AND DSP STACKS FOR PS-256-QAM TRANSMISSION

The experimental setup for single-channel transmission is shown in Supplementary Fig. S2a. At the transmitter, we choose two lasers for the signal and LO from 100-kHz ECL (EXFO IQS-636), 1-MHz DFB and 3-MHz DFB. The dual-polarization signal is generated using two 27-GHz 3-dB bandwidth single-polarization in-phase/quadrature modulators (SP IQ Mod. 1/2). They are driven by the dual-subcarrier 45-Gbaud PS-256-QAM electrical signal generated using a 120-GSa/s arbitrary waveform generator (AWG, Keysight M8194) with a 3-dB bandwidth of 45 GHz. After combined with a polarization beam combiner (PBC), the signal is launched to the 80-km standard single-mode fiber (SSMF) link or directly sent to the coherent receiver. For the back-to-back scenario, the optical signal-to-noise ratio (OSNR) is adjusted through a variable optical attenuator (VOA) followed by optical amplification. For 80-km SSMF transmission, another erbium-doped fiber amplifier (EDFA) is placed to optimize the launch power.

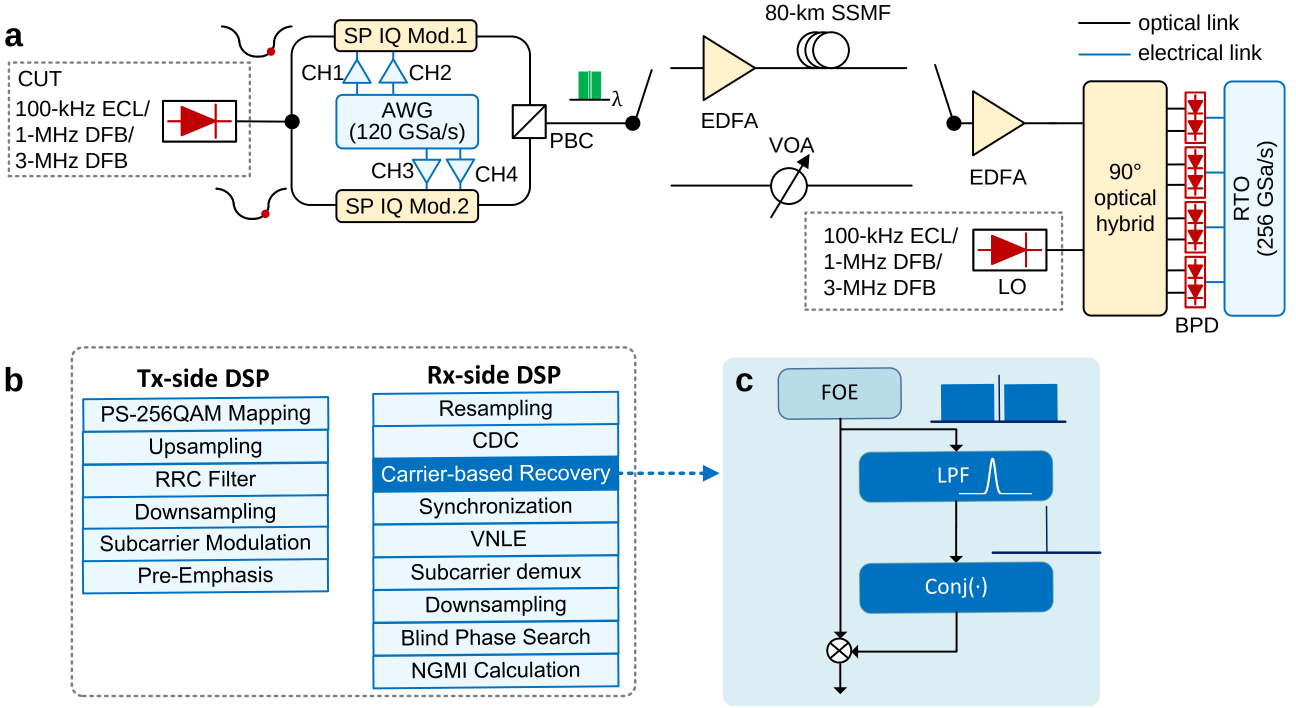

Fig. S2. The experimental setup and DSP stacks for the single-channel PS-256-QAM signal transmission. a The experimental setup for single-channel PS-256-QAM signal transmission with different laser linewidth configurations. **b** The transmitter- and receiver-side digital signal processing (DSP) stacks. Tx, transmitter; Rx, receiver; RRC, root raised cosine; CDC, chromatic dispersion compensation; VNLE, Volterra nonlinear equalizer. **c** The residual carrier-based frequency offset removal and phase recovery. FOE, frequency offset estimation; LPF, low-pass filter; Conj, conjugate.

At the receiver, after amplified by the EDFA to control the received optical power, the signal is sent to the 90° optical hybrid and detected by the balanced photodiodes (BPD, Finisar BPDV3120R) with a 3-dB bandwidth of 70 GHz. Finally, the electrical signal is captured for offline signal processing using the 256-GSa/s real-time oscilloscope (RTO, Keysight UXR0594AP).

The digital signal processing (DSP) procedures are shown in Fig. S2b. At the transmitter, the data is mapped to 40960 PS-256-QAM symbols first, which is framed by a 4352-symbol preamble for synchronization and channel equalization. After 8 times up-sampling, the signal is pulse-shaped with a roll-off factor of 0.05. Then the signal is re-sampled to match the AWG sampling rate. Subsequently, a dual-band subcarrier modulation is conducted with an optimized guard band of 2.0 GHz. A linear pre-emphasis [2] is applied to compensate for the transmitter bandwidth limitation.

At the receiver, we first re-sample the captured signal to 4 samples-per-symbol (SPS). Then we apply chromatic dispersion compensation (CDC) for 80-km SSMF transmission. Subsequently, the frequency offset estimation (FOE) and carrier phase recovery are completed with the aid of the residual carrier, as shown in Fig. S2c. After frame synchronization, the channel equalization is performed by 3rd-order sparse Volterra nonlinear equalizer (VNLE). The 1st-, 2nd-, and 3rd-order memory lengths of the VNLE are optimized to 161, 41, and 11, respectively. After subcarrier demultiplexing and down-sampling to 1 SPS, we use the blind phase search (BPS) algorithm [3] to finely correct the

residual phase fluctuation within $\pm 5.3^\circ$. Then the GMI or NGMI is calculated to evaluate the performance.

NOTE 3 - DETAILED SIMULATION SETUP FOR OSNR PENALTY COMPARISON

The simulation is accomplished in the commercial software VPItransmissionMaker 10.1. The setup is shown in Fig. **S3**, where the optical signal-to-noise power (OSNR) is controlled using the 'Set OSNR' module. The detailed simulation parameters are displayed in Table. **S1**. By changing the direct current voltage of the modulator, the operation point can be altered from the null point in the conventional time-domain pilot setup to the residual carrier modulation setup.

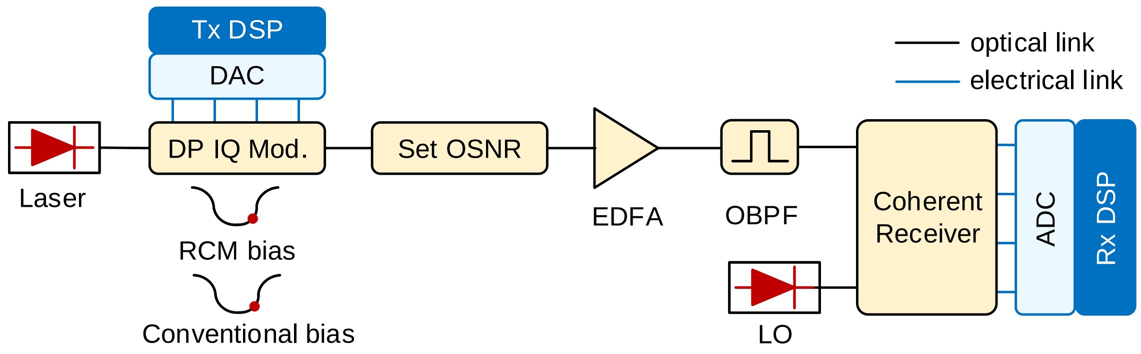

Fig. S3. The simulation setup in the VPItransmissionMaker 10.1 software. Here the operation point of the modulator is also shown for the residual carrier setup and the conventional time-domain pilot-based phase recovery setup. DSP, digital signal processing; DP IQ Mod., dual-polarization in-phase/quadrature modulator; OSNR, optical signal-to-noise ratio; EDFA, erbium-doped fiber amplifier; OBPF, optical band-pass filter; LO, local oscillator.

Table S1. The parameters of the simulation system

**Parameters**	**RCM**	**TP**
**Baud rate**	2 subcarriers × 45 GBaud	45 GBaud
**Modulation format**	PS-256-QAM	PS-256-QAM
**Extinction ratio of modulators**	50 dB	50 dB
$\mathbf{V}_\pi$	5 V	5 V
**Signal laser frequency**	193.1 THz	193.101 THz
**DAC/ADC resolution**	8 bits	8 bits
**Optical filter bandwidth**	108 GHz	54 GHz

RCM, residual carrier modulation; TP, time-domain pilot-based phase recovery.

NOTE 4 - DETAILED EXPERIMENTAL SETUP AND DSP STACKS FOR 512-QAM ANALOG RADIO-OVER-FIBER TRANSMISSION

Figure S4 shows the experimental setup for high-fidelity 512-QAM analog radio-over-fiber transmission. For the channel under test (CUT), we use a 100-kHz linewidth external cavity laser (ECL, EXFO IQS-636) as the optical source. A 120-GSa/s arbitrary waveform generator (AWG, Keysight M8194) produces the electrical 17-Gbaud 512-QAM waveform, which drives a 27-GHz single-polarization in-phase/quadrature modulator (SP IQ Mod.). The bias point is slightly above the null point to introduce a residual carrier with ∼ -15-dB carrier-to-signal power ratio (CSPR). Polarization-division-multiplexing (PDM) is emulated by a split-and-decorrelation structure. For the loading channels, 11 ECLs spacing at 50 GHz are combined by a polarization-maintaining optical coupler (PM-OC) and modulated by another IQ modulator. We then place a wavelength-selective switch to merge and flatten the CUT and loading channels. The transmission link is 10-km standard single-mode fiber (SSMF) to cover the typical range of the fiber-wireless access network.

At the receiver, we employ an optical band-pass filter (OBPF) to demultiplex the target wavelength channel. After optical amplification, the signal beats with the 100-kHz linewidth local oscillator (LO, EXFO IQS-636) in the 90° optical hybrid and is detected by four 70-GHz balanced photodiodes (BPD, Finisar BPDV3120R). The electrical waveforms of the in-phase and quadrature components on both polarizations are captured by a 256-GSa/s real-time oscilloscope (RTO, Keysight UXR0594AP) for offline processing.

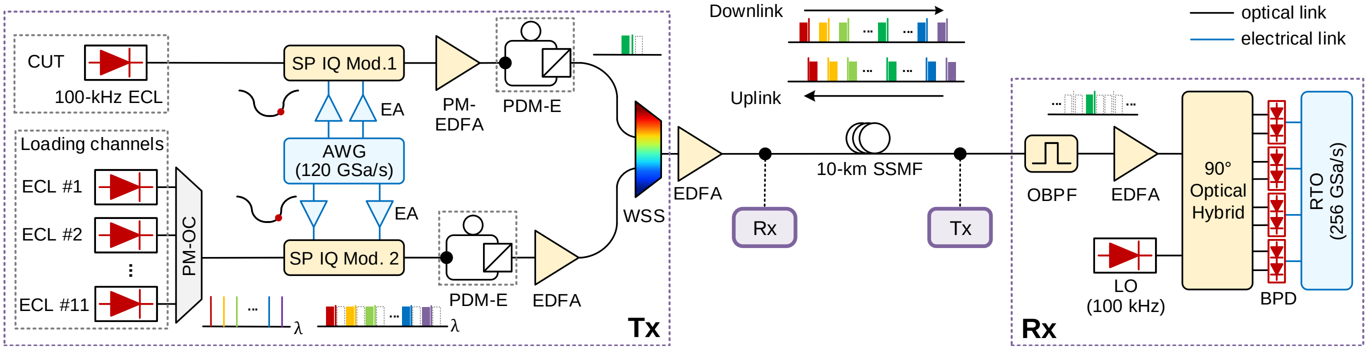

Fig. S4. Experimental setup of 12-channel wavelength-division-multiplexed (WDM) 512-QAM analog radio-over-fiber transmission system. Tx, transmitter; Rx, receiver; CUT, channel under test; ECL, external cavity laser; SP IQ Mod., single-polarization in-phase/quadrature modulator; EA, electrical amplifier; AWG, arbitrary waveform generator; PM-EDFA, polarization-maintaining erbium-doped fiber amplifier; PDM-E, polarization-division-multiplexing emulator; WSS, wavelength-selective switch; SSMF, standard single-mode fiber; OBPF, optical band-pass filter; LO, local oscillator; BPD, balanced photodiode; RTO, real-time oscilloscope.

Figure S5 displays the transmitter- and receiver-side digital signal processing (DSP) stacks. At the transmitter, we generate 32768 data symbols with the modulation format of 512-QAM as the payload. We insert 3072 symbols in front of the payload as the preamble. After up-sampling, the symbol sequence convolutes with a digital root-raised cosine (RRC) filter with a roll-off of 0.1. In the presence of IQ imbalance, we up-convert the baseband signal to separate the signal from its conjugation replica. Specifically, we use left and right sideband modulation for the downlink and uplink, respectively, which also suppresses the backscattering. We reserve a 1.0-GHz guard band between the up-converted signal and the residual carrier to avoid the crosstalk. Then the signal is re-sampled to match the sampling rate of AWG. Before sending to the AWG, we perform linear pre-emphasis [2] in the frequency domain to compensate for the transmitter bandwidth limitation.

At the receiver, we first correct the relative skew values among the four channels of the RTO. We then re-sample the electrical waveform to 3 samples-per-symbol (SPS) to avoid spectral overlap. In the carrier-based recovery stage, the frequency offset is estimated by the peak position in the frequency domain, while the carrier phase recovery is implemented by a low-pass filter, conjugation, and digital beating. For frame synchronization, we calculate the cross-correlation between the transmitted and received preamble. A 4×2 multi-input-multi-output (MIMO) sparse Volterra nonlinear equalizer is employed to realize polarization demultiplexing, fiber dispersion compensation, and transceiver distortion mitigation simultaneously. The tap coefficients are updated by the recursive least square (RLS) algorithm for fast convergence. The tap lengths are 81, 11, and 11 for the 1st-, 2nd-, and 3rd-order kernels, respectively. Afterwards, the signal is down-converted to the baseband and down-sampled to 1 SPS. We use the blind phase search (BPS) algorithm [3] to finely correct the residual phase noise within ±8°. Finally, we evaluate the signal quality by calculating the bit-error rate (BER) and the recovered signal-to-noise ratio (SNR).

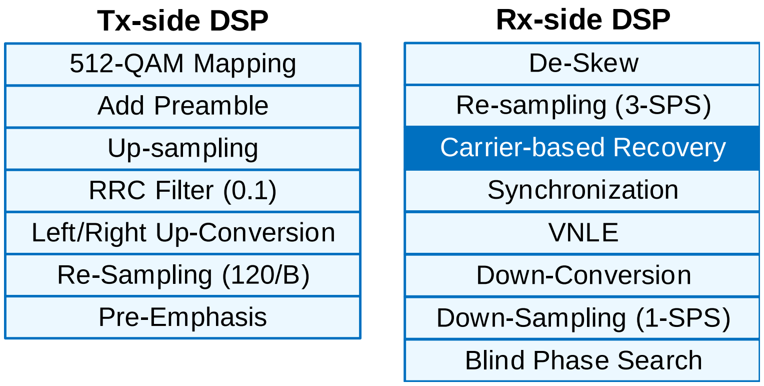

Fig. S5. Transmitter and receiver-side DSP stacks. RRC, root-raised cosine; B, baud rate; SPS, sample-per-symbol; VNLE, Volterra nonlinear equalizer.

NOTE 5 - THE THEORETICAL DEPENDENCE OF EFFECTIVE OSNR ON THE CSPR

For conventional self-coherent systems, a strong carrier is required to eliminate the signal-signal beating interference (SSBI) [4, 5]. Consequently, this leads to a deterioration of the effective optical signal-to-noise ratio (OSNR) for the information-bearing signal. In other word, the OSNR is no longer a fair metric because it contains both the optical power of the signal and the unsuppressed carrier. For clear distinction, we use OSNR_{eff} to denote the effective OSNR, where the signal power refers to the total signal power without the carrier. According to the definition, the relationship between the OSNR_{eff} and CSPR can be derived as

$$\text{OSNR} = \frac{P_{\text{signal}} + P_{\text{carrier}}}{P_{\text{noise}}} = \frac{P_{\text{signal}} \cdot (1 + \text{CSPR})}{P_{\text{noise}}} = \text{OSNR}_{\text{eff}} \cdot (1 + \text{CSPR})$$

Here P_{signal}, P_{carrier}, and P_{noise} denote the optical power of the signal, carrier, and the noise, respectively. As shownn in Fig. **S6**, the effective OSNR will decreases as the CSPR increases. However, when the CSPR is below -10dB, the impact of the reduced effective OSNR is marginal. For a CSPR value of -11.4 dB used in the PS-256-QAM signal transmission, the reduced effective OSNR can be calculated as

$$\Delta\text{OSNR}_{\text{eff}} = 10\text{lg}\left(\frac{1}{1 + \text{CSPR}}\right) = 10\text{lg}\left(\frac{1}{1 + 10^{-11.4/10}}\right) = -0.3 \text{ dB}$$

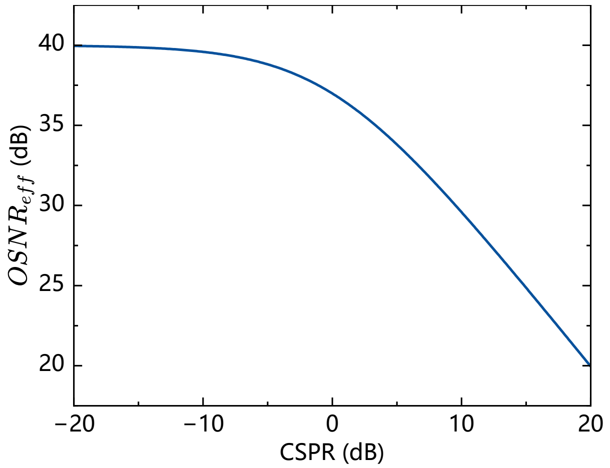

Fig. S6. The effective OSNR as a function of the CSPR when the OSNR is set to be 40 dB.

NOTE 6 - THE TRANSMISSION PERFORMANCE WITH DIFFERENT SOURCE ENTROPY

As illustrated in Fig. S7, we measure the NGMI for PS-256-QAM signal with different source entropy in the single-channel 80-km SSMF transmission system. For a source entropy of 12.82 bits/4D-symbol, a NGMI of 0.9226 is obtained. Compared to the WDM case, the NGMI in the single-channel configuration is higher by 0.022, corresponding to a GMI difference of 0.352 bits/4D-symbol.

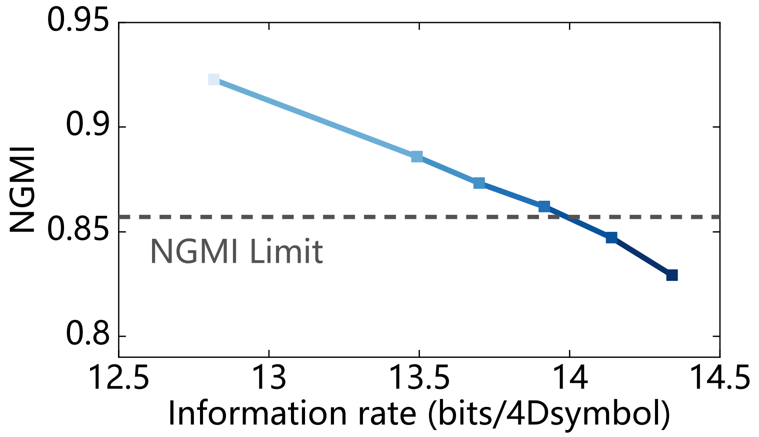

Fig. S7. Measured NGMI versus different source entropy values for single-channel 80-km SSMF transmission.

NOTE 7 - COMPARISON BETWEEN RCM AND TIME-DOMAIN OR FREQUENCY-DOMAIN PILOT BASED PHASE RECOVERY

1. Advantages of RCM over time-domain pilot (TP)

(1) Continuous Phase Tracking: In scenarios with significant phase noise and no additive white Gaussian noise (AWGN), RCM enables continuous phase tracking, whereas TP schemes can only track pilot symbols periodically at intervals of several dozen symbols. Even with linear interpolation for intermediate values, TP cannot accurately estimate phase at intermediate times. Our simulations reveal that RCM provides more accurate phase noise estimation, as shown in Fig. S8. In simulation, the true phase noise and the phase noise estimated by time-domain pilot or residual carrier, is shown in Fig. S8(a) and Fig. S8(b). The Tx- and Rx-side laser linewidth sum is 3 MHz. We use the root mean square error (RMSE) to quantify the deviation between the estimated phase noise and the true phase noise. RMSE is defined as

$$\mathrm{RMSE} = \sqrt{\frac{1}{N}\sum_{n=1}^{N}\left|\hat{\varphi}(n) - \varphi(n)\right|^2}.$$

Here $\hat{\varphi}(n)$ is the estimated phase noise by RCM or TP. $\varphi(n)$ is the true phase noise value, which is obtained through dividing received signal by transmitted signal.

The calculated RMSE for RCM is 3.54°. In comparison, the calculated RMSE for TP method is 8.85°. Even with linear interpolation, the RMSE for TP remains relatively high at 8.05°.

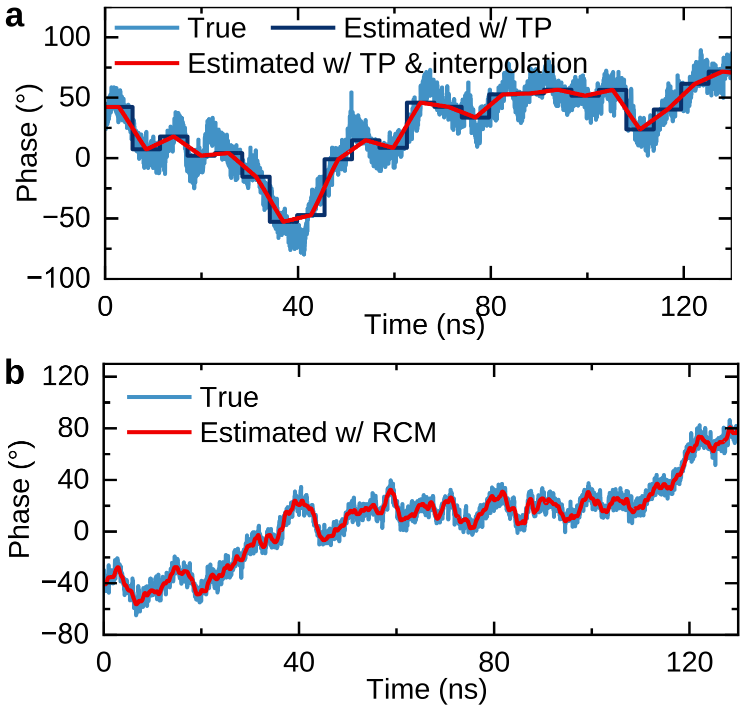

Fig. S8. The true phase noise and estimated phase noise by (a) TP or (b) RCM.

(2) Phase SNR Advantage: In the presence of both phase noise and AWGN, the RCM scheme employs a low-pass filter in the phase information extraction process, offering a signal-to-noise ratio (SNR) advantage for phase information estimation. This reduces the impact of mistaking additive noise for phase noise on phase recovery. For RCM, the SNR of the phase information can be described as:

$$\mathrm{SNR_{RCM}} = \frac{P_\mathrm{S}}{P_\mathrm{N}} = \frac{P_\mathrm{C}}{S(f)\cdot B_\mathrm{T}}.$$

Here P_S is the power of the phase information signal. P_N is the AWGN noise. P_C is the power of the residual carrier at the receiver. $S(f)$ is the power spectral density (PSD) of the noise. B_T is the low-pass filter bandwidth in RCM. For traditional time-domain pilot-based phase recovery with a signal bandwidth of B, the SNR of the phase information

can be described as

$$\mathrm{SNR_{TP}} = \frac{P_\mathrm{S}}{P_\mathrm{N}} = \frac{P_\mathrm{S}}{S(f)\cdot B}.$$

Then, it follows that

$$\frac{\mathrm{SNR_{RCM}}}{\mathrm{SNR_{TP}}} = \frac{P_\mathrm{C}}{P_\mathrm{S}}\cdot\frac{B}{B_\mathrm{T}} = \mathrm{PTSPR}\cdot\frac{B}{B_\mathrm{T}}.$$

It can be seen that as long as the ratio of the low-pass filter bandwidth to the signal bandwidth is less than the pilot tone to signal power ratio (PTSPR), RCM achieves an SNR advantage. In the early days of digital coherent transmission, the systems are with low baud rate, so the condition where the low-pass filter bandwidth to signal bandwidth ratio is less than PTSPR may not be satisfied, and the performance of RCM and TP may be similar. However, in high-speed systems, the RCM can significantly outperform TP. For example, the PTSPR is about -13 dB in Fig. 5(c), B is around 95 GHz, B_T is 360 MHz, we can get

$$\frac{\mathrm{SNR_{RCM}}}{\mathrm{SNR_{TP}}} = 10^{(-13/10)}\cdot\frac{95\times10^9}{360\times10^6} = 13.2 > 1$$

Thus, the condition for SNR advantage holds true.

2. Advantages of RCM over RF pilot tone-based phase recovery

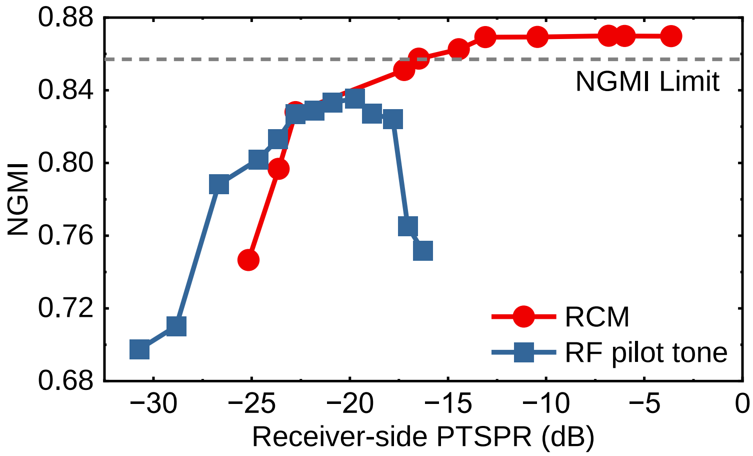

Fig. S9. Measured NGMI versus the receiver-side PTSPR for RCM and RF pilot tone schemes

Pilot tone in the frequency domain is another way to realize carrier phase recovery. It is also referred as RF pilot tone, or digital pilot tone equivalently. The proposed RCM scheme distinguishes itself from the RF pilot tone because it is generated in the optical domain, which does not occupy precious DAC quantization bits.

To reveal the advantages, we provide a more comprehensive comparison between RF pilot tone and RCM. In the experiment, we used a 3-MHz laser as the signal laser and a 100-kHz laser as the local oscillator. The results are shown in Fig. R5. For a fair comparison, pilot tone to signal power ratio (PTSPR) at the receiver side is employed as the metric for the power of the pilot tone or residual carrier, which is defined as

$$\mathrm{PTSPR} = 10\times\log_{10}\left(\frac{P_\mathrm{pilot}}{P_\mathrm{signal}}\right)$$

Here P_pilot and P_signal are the power of the pilot tone component and the signal in electrical spectrum at the receiver, respectively.

When the PTSPR of the RF pilot tone is large enough, the performance can be close to that of residual carrier modulation, as shown in Fig. S9 for PTSPR around -20 dB. However, since the RF pilot tone requires generating digital pilot tone components at the transmitter through the DAC, it occupies precious DAC quantization bits. Further increasing the PTSPR will increase quantization noise, thereby degrading system performance. In contrast, the residual carrier scheme can further improve performance by increasing the PTSPR. A much better performance

with RCM can be observed when the receiver PTSPR is above -13 dB, compared to RF pilot tone scheme.

Moreover, we also measured the signal performance under different modulation formats with the same experiment setup. As shown in Fig. S10, for low-order modulation formats such as 16-QAM, the RF pilot tone can get almost the same phase noise compensation performance as RCM scheme. However, for high-order modulation signals, RCM can get a better performance. For example, a GMI improvement of 0.45bit/4D-symbol can be obtained for PS-256-QAM with an entropy of 13.92 bit/4D-symbol.

It is worth noting that our experiment used DACs with 8-bits vertical resolution and an effective number of bits (ENOB) of about 5.5 bits (Keysight 8194A). In practical applications, the resolution of the DACs would likely be lower, making the advantage of RCM even more apparent.

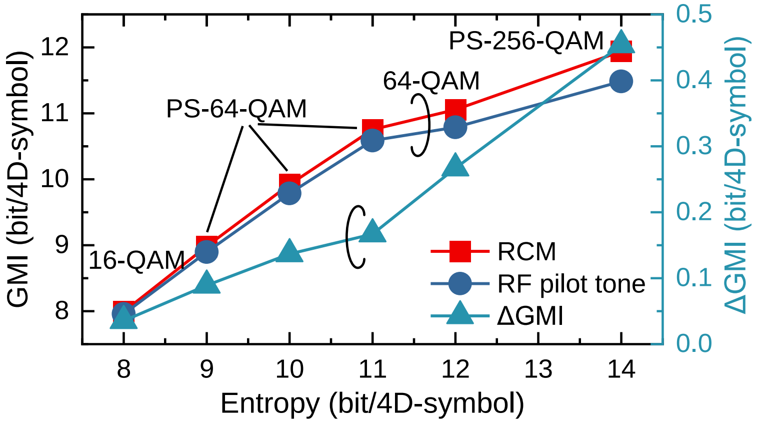

Fig. S10. Measured GMI and GMI difference versus the transmitted signal entropy for RCM and RF pilot tone schemes.

NOTE 8 - LINEAR AND NONLINEAR EQUALIZATION PERFORMANCE IN RCM

Deviation from the null point of an IQ modulator may lead to a decrease in modulation linearity. In this regard, we compare the NGMI result with linear equalizer only and with Volterra nonlinear equalizer (VNLE) in Fig. **S11**. The equalizer taps of different systems are optimized separately to ensure a fair comparison. Additionally, the NGMI difference between linear and Volterra nonlinear equalization of RCM is also shown in Fig. **S12**. It shows that the NGMI difference between linear equalization and Volterra nonlinear equalization is quite small. This observation may relate to the relatively low PTSPR of the RCM and also emphasize the advantage of RCM.

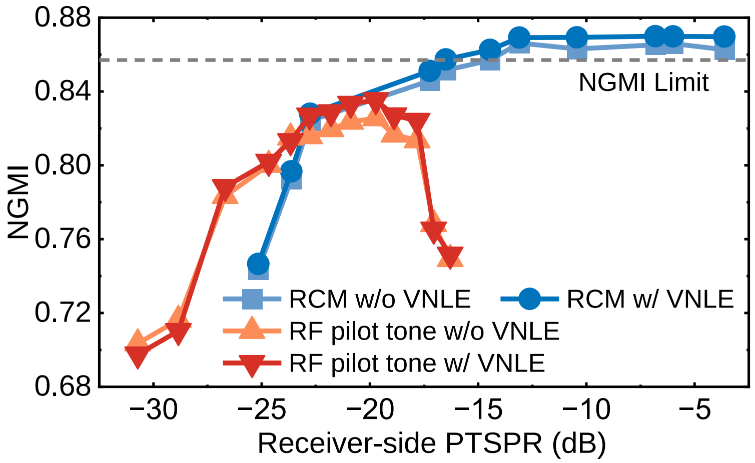

Fig. S11. Measured NGMI versus receiver-side PTSPR for RCM and RF pilot tone schemes with (w/) and without (w/o) VNLE.

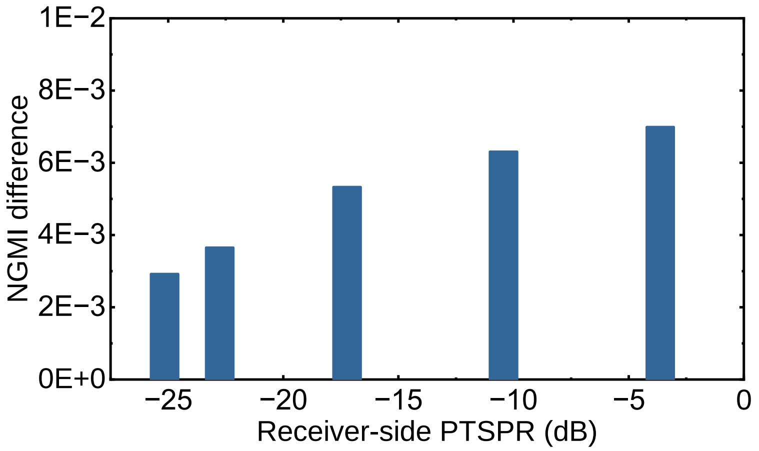

Fig. S12. Measured NGMI difference versus receiver-side PTSPR for RCM and RF pilot tone schemes with and without VNLE.

NOTE 9 - POLARIZATION DISTORTION AND ITS INFLUENCE ON RCM

The CSPR of the residual carrier on each polarization may fluctuate with polarization rotation in the fiber link due to the interference between the residual carrier components between orthogonal polarizations. In our manuscript, we address this issue by recovering the phase on the residual carrier at the polarization with a higher CSPR. Here we name it selection combining method (SC). Similar to wireless communications, besides the SC we used, other methods such as equal gain combining (EGC) and maximum ratio combining (MRC) can also be utilized for combining the phase information from residual carriers on two orthogonal polarizations.

(1) Selection combining method: Select the residual component that has higher CSPR in the two polarizations.

(2) Equal gain combining method: Adds the two residual components with equal weights.

(3) Maximum ratio combining method: Adds the two residual components with different weights, proportional to their CSPR.

For the EGC and MRC, when a phase difference of occurs between the residual carrier components between orthogonal polarizations due to the polarization rotation, the fixed phase difference is detected and compensated first before combining.

We conduct simulations to compare the performance of these three strategies. Polarization rotation is simulated by changing the rotation of state of polarization (RSOP) as follows [6]:

$$\begin{bmatrix} E_{\mathrm{x,out}} \\ E_{\mathrm{y,out}} \end{bmatrix} = \begin{bmatrix} \cos\theta & e^{-j\phi}\sin\theta \\ -e^{j\phi}\sin\theta & \cos\theta \end{bmatrix} \begin{bmatrix} E_{\mathrm{x,in}} \\ E_{\mathrm{y,in}} \end{bmatrix}$$

Here $E_{\mathrm{x,in}}$ and $E_{\mathrm{y,in}}$ is the Jones vector components of the optical field at the transmitter. $E_{\mathrm{x,out}}$ and $E_{\mathrm{y,out}}$ is the Jones vector components at the receiver.

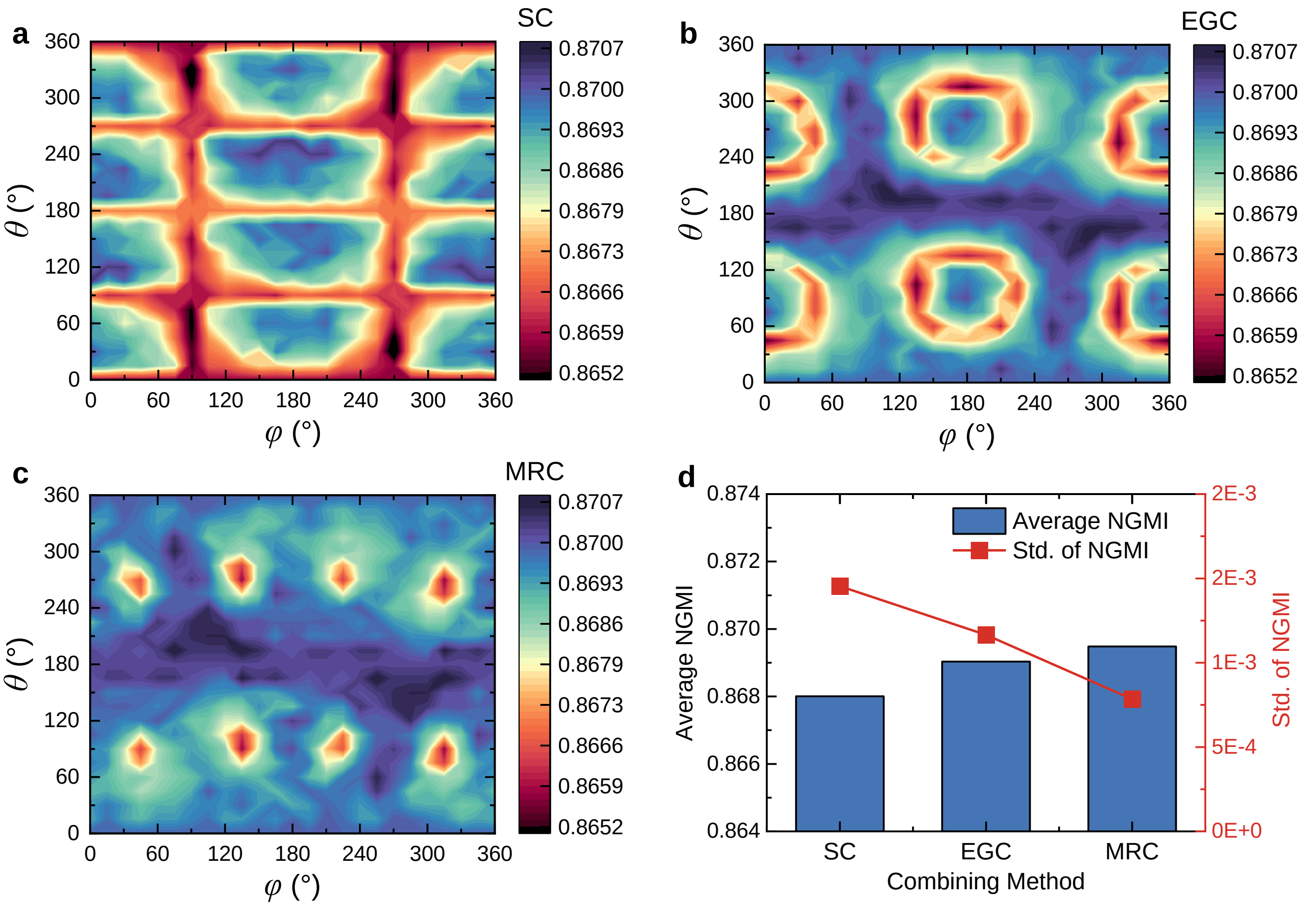

Fig. S13. Measured NGMI under different RSOP with (a) selection combining (SC), (b) equal gain combining (EGC), (c) maximum ratio combining (MRC) strategies. (d) The averaged NGMI and standard deviation of NGMI with different combining strategies.

The simulation system uses a 3-MHz linewidth laser as the signal laser and a 100-kHz linewidth laser as the LO. The transmitted signal is dual-subcarrier 45-GBd PS-256-QAM, consistent with the manuscript.

As shown in Fig. **S13**(a)-(c), the measured NGMI is observed to be stable under all different RSOPs, and the three strategies exhibit similar performance. The mean value and standard deviation of measured NGMI using different combining strategies are shown in Fig. **S13**(d). It can be seen that MRC can get slightly best performance. But the difference is quite small.

Regarding the Polarization Mode Dispersion (PMD) effect, the standard single-mode fiber has a PMD coefficient of 0.2 ps/sqrt(km). Even without considering polarization rotation (the worst case), the PMD-induced differential delay at 80 km is 1.79 ps. Additionally, since phase noise is narrowband (typically several hundred MHz), this delay can be considered as introducing phase modulation $\varphi = \exp(j2\pi f\tau)$ on a particular polarization. Therefore, the impact of this delay can be ignored.

NOTE 10 - BIAS CONTROL METHOD FOR GENERATING THE RESIDUAL CARRIER

In our experiment, we manually adjust the bias voltage to generate a residual carrier with a target pilot tone to signal power ratio.

From the perspective of practical use, the most common auto-bias control methods for high-order modulation formats are the dither signal detection techniques [7–9]. These methods introduce small, low-frequency sine or square waves as the dither signal into the bias pin of the modulator, and adjust the bias voltage automatically according to the output optical signal. By using dither-correlation detection and a specifically designed dither signal loading scheme [9], precise and stable arbitrary bias point control can be achieved. Therefore, this technique is feasible to maintain a stable bias voltage in residual carrier modulation systems.

[1] Ludvigsen, H., Tossavainen, M. & Kaivola, M. Laser linewidth measurements using self-homodyne detection with short delay. *Optics Communications* **155**, 180–186 (1998).

[2] Zou, D. *et al.* Comparison of bit-loading DMT and pre-equalized DFT-spread DMT for 2-km optical interconnect system. *Journal of Lightwave Technology* **37**, 2194–2200 (2019).

[3] Pfau, T., Hoffmann, S. & Noe, R. Hardware-Efficient Coherent Digital Receiver Concept With Feedforward Carrier Recovery for M-QAM Constellations. *Journal of Lightwave Technology* **27**, 989–999 (2009).

[4] Mecozzi, A., Antonelli, C. & Shtaif, M. Kramers–Kronig coherent receiver. *Optica* **3**, 1220–1227 (2016).

[5] Shieh, W., Sun, C. & Ji, H. Carrier-assisted differential detection. *Light: Science & Applications* **9**, 18 (2020).

[6] Savory, S. J. Digital coherent optical receivers: Algorithms and subsystems. *IEEE Journal of selected topics in quantum electronics* **16**, 1164–1179 (2010).

[7] Kawakami, H., Kobayashi, T., Yoshida, E. & Miyamoto, Y. Auto bias control technique for optical 16-qam transmitter with asymmetric bias dithering. *Optics Express* **19**, B308–B312 (2011).

[8] Zhu, X. *et al.* Coherent detection-based automatic bias control of mach–zehnder modulators for various modulation formats. *Journal of Lightwave Technology* **32**, 2502–2509 (2014).

[9] Li, X. *et al.* Arbitrary bias point control technique for optical iq modulator based on dither-correlation detection. *Journal of Lightwave Technology* **36**, 3824–3836 (2018).
